# Use of antibiotics by spanish dentists receiving postgraduate training in endodontics

**DOI:** 10.4317/jced.54894

**Published:** 2018-07-01

**Authors:** Oscar Alonso-Ezpeleta, Milagros Martín-Jiménez, Benjamín Martín-Biedma, José López-López, Leopoldo Forner-Navarro, Jenifer Martín-González, Paloma Montero-Miralles, María del Carmen Jiménez-Sánchez, Eugenio Velasco-Ortega, Juan J. Segura-Egea

**Affiliations:** 1 Department of Endodontics, School of Health Sciences, University of Zaragoza, Spain; 2 Department of Endodontics, School of Dentistry, University of Sevilla, Spain; 3 Department of Endodontics, School of Medicine and Dentistry, University of Santiago, Spain; 4 Department of Oral Medicine, School of Medicine and Dentistry, University of Barcelona, Spain; 5 Department of Oral Medicine, School of Medicine and Dentistry, University of Valencia, Spain; 6 Department of Comprehensive Dentistry, School of Dentistry, University of Sevilla, Spain

## Abstract

**Background:**

The incidence of endodontic infections is high. The contribution of Endodontics to the global problem of antibiotic resistance could be significant. The ESE, together with the World Health Organization, are promoting the World Antibiotic Awareness Week (13-19 November 2017) to promote the appropriate use of systemic antibiotics in Endodontics. The objective of this study was to determine the prescription pattern of antibiotics in the treatment of endodontic infections of Spanish dentists attending specialization programs in Endodontics.

**Material and Methods:**

Dentists from five Spanish endodontic postgraduate programs were requested to answer a one-page questionnaire surveying about antibiotics indications. Seventy-three dentists were required to participate in this investigation, and 67 (91.2%) fulfilled satisfactorily the survey and were included in the study. Data were analyzed using descriptive statistics and chi square test.

**Results:**

The average duration of antibiotic therapy was 6.8±1.2 days. All respondents chose amoxicillin as first choice antibiotic in patients with no medical allergies, alone (40%) or associated to clavulanic acid (60%). The first drug of choice for penicillin allergic patients was clindamycin (72%). For cases of irreversible pulpitis, 22% of respondents prescribed antibiotics. For the scenario of a necrotic pulp, symptomatic apical periodontitis and no swelling, 37% prescribed antibiotics. A quarter of dentists prescribed antibiotics for necrotic pulps with asymptomatic apical periodontitis and a sinus tract.

**Conclusions:**

The results of this study show that postgraduate training in Endodontics provides greater awareness of the correct indications of antibiotics. Dentists who have received specialized training in Endodontics have a prescription pattern of antibiotics more adjusted to the guidelines recommended by international organizations and by scientific societies.

** Key words:**Antibiotics, apical periodontitis, dental curriculum, endodontic infections, postgraduate endodontic training.

## Introduction

The need for expertise in Endodontics should be met through a harmonized effort in development of basic educational programmes at undergraduate level and, furthermore, programmes at postgraduate levels (1). The ESE undergraduate curriculum guidelines for Endodontology state that a graduating European dentist should have knowledge of pharmacology and therapeutics applied to the management of patients suffering endodontic infections (2). Therefore, the dental curriculum in European universities includes pharmacology, involving the study of antimicrobial drugs.

At postgraduate level, endodontic trainees must have an understanding of the relevant aspects of the clinical pharmacology and therapeutics in relation to the treatment of endodontic infections (1). Consequently, the programs of postgraduate studies of Endodontics should include that scientific evidence shows that antibiotics are not indicated for the treatment of irreversible pulpitis, or for pulpal necrosis, nor for cases of localized acute apical abscesses (3-7). It is especially important that postgraduate students in Endodontics know that there is no evidence to support the indication of systemic antibiotic therapy to relieve pain in cases of irreversible pulpitis (8).

However, different surveys have shown that Spanish (9,10), European (11-13) and also worldwide (14), dentists follow inadequate prescribing patterns of systemic antibiotics in the treatment of endodontic infections. In the recently published study by Martín-Jiménez *et al.* (15) analysing dental students’ knowledge regarding the indications for antibiotics in the management of endodontic infections, has shown that nearly 50% of the students of the last year of the degree of dentistry would apply inappropriate antibiotic guidelines in the treatment of endodontic infections. The results of this study show that up to 63%, 44% and 40% of students would prescribe antibiotics in cases of irreversible pulpitis, necrotic pulp with symptomatic apical periodontitis and no swelling, and necrotic pulps with asymptomatic apical periodontitis and a sinus tract, respectively. None of these three clinical situations requires treatment with antibiotics (7).

Considering the high prevalence of endodontic infections (16-18) and the relatively high impact (10%) of dentists in the total antibiotic prescription (19,20), the contribution of Endodontics to the serious global problem of antibiotic resistance could be significant. Actually, the ESE, together with the World Health Organization, are promoting the World Antibiotic Awareness Week (13-19 November 2017) to promote the appropriate use of local and systemic antibiotics in Endodontics and prevent their misuse.

Dentists who attend endodontic specialization programs should be better aware of the indications of systemic antibiotic therapy for the treatment of endodontic diseases. However, no study has analysed the knowledge and the prescription pattern of postgraduate dentists in Endodontics about the use of antibiotics in the treatment of endodontic infections.

The objective of this study was to determine the prescription pattern of Spanish dentists attending specialization programs in Endodontics on the indication of systemic antibiotic therapy in the treatment of endodontic infections.

## Material and Methods

Ethical approval was sought, and ethical guidelines were followed. The Ethics Committee of the University of Seville approved the study protocol.

-Study population 

Dentists attending specialization programs in Endodontics from five Endodontic Postgraduate Programs developed in five different Spanish Dental Schools (Barcelona, Santiago, Sevilla, Valencia y Zaragoza), were requested to answer a one-page questionnaire on the indications for systemic antibiotics in the treatment of endodontic infections.

-Questionnaire

**Figure 1:**
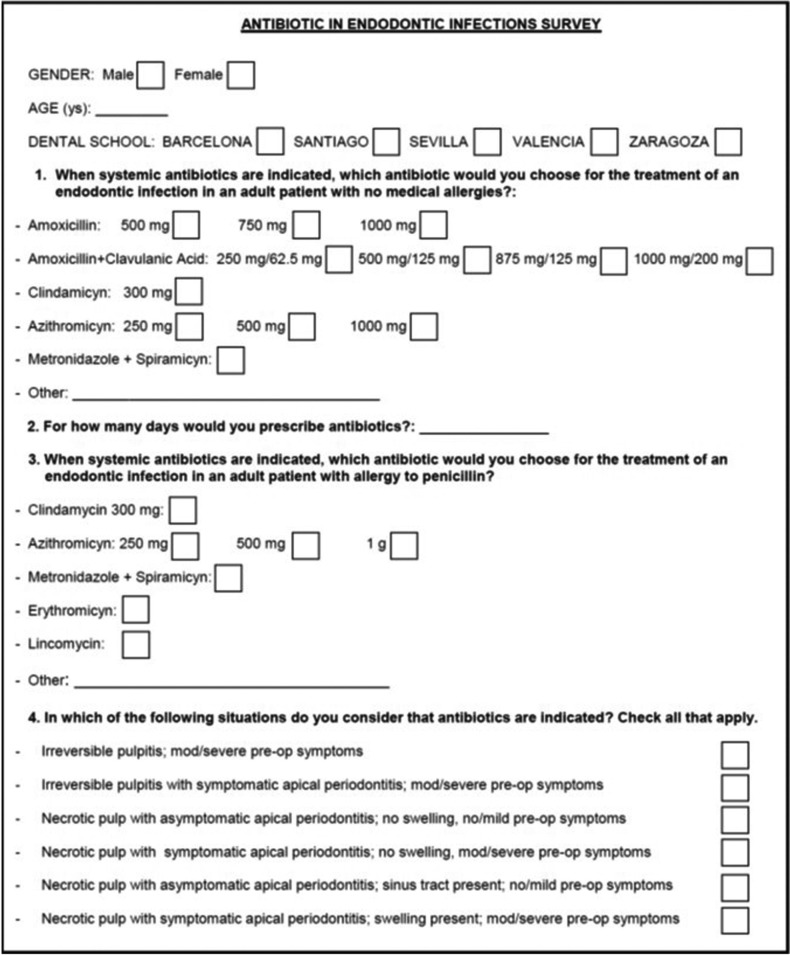
Antibiotic survey.

The questions (Fig. 1) were based on those asked in the previous surveys developed in USA (21,22) and Spain (9,10,15). The pulpal and periapical diagnoses were as follows: 1) Irreversible pulpitis with moderate/severe symptoms. 2) Irreversible pulpitis with symptomatic apical periodontitis and moderate/severe symptoms. 3) Necrotic pulp with asymptomatic apical periodontitis, no swelling, and no or mild symptoms. 4) Necrotic pulp with symptomatic apical periodontitis, no swelling, and moderate/severe symptoms. 5) Necrotic pulp with asymptomatic apical periodontitis, sinus tracts and no/mild symptoms. 6) Necrotic pulp with symptomatic apical periodontitis, swelling, and moderate to severe symptoms. One trained professor of each Endodontic Postgraduate Program administered the questionnaires to the dentists, who participated voluntarily, anonymously and without compensation.

-Data collection and statistical analysis.

A database was created for further analysis using Excel (Microsoft Corp., Redmond, WA, USA). Data description was carried out by frequency tables. When obtaining the numerical representation by percentages, the total number of answers for each query was taken into account. Data were analysed using descriptive statistics and chi square test. ANOVA and the Tukey test for independent samples were used to assess differences between dental schools. Significant differences were considered when *p* < 0.05.

## Results

-Participation and profile of respondents

Seventy-three dentists were required to participate in the investigation and 67 (91.2%) (University of Barcelona, n = 13; University of Santiago de Compostela, n = 6; University of Sevilla, n = 19; University of Valencia, n = 14; and University of Zaragoza, n = 15) fulfilled satisfactorily the survey and were included. The demographics of the 67 respondents are described in  Table 1. Male respondents (n =34) accounted for 51% and females (n = 33) 49% of the total. The mean age of the respondents was 30.0 years (SD = 5.7).

**Table 1:**
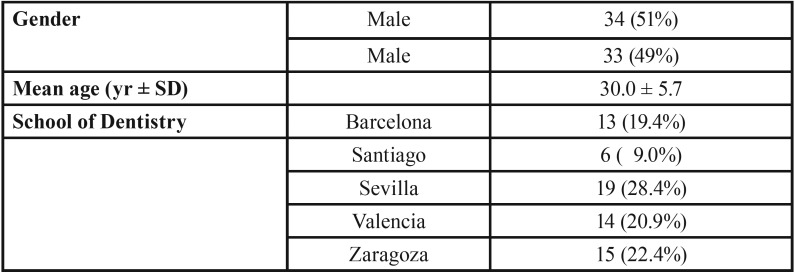
Description of respondents (n = 67).

-Duration of antibiotic treatment

**Figure 2:**
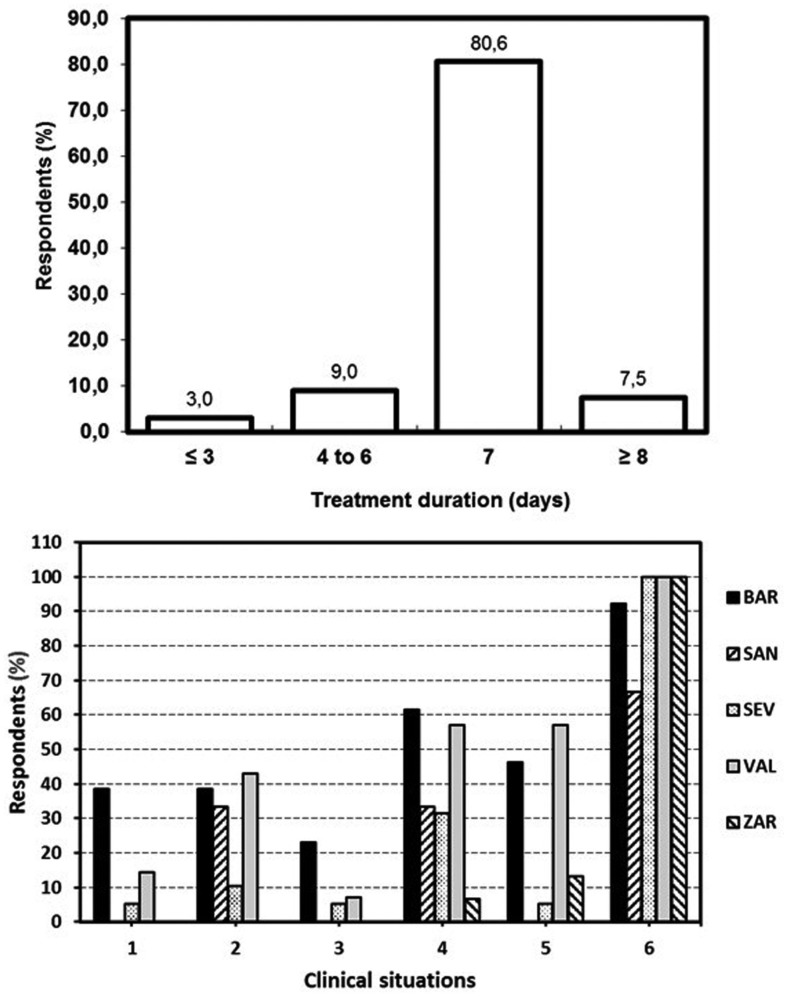
Top. Distribution of respondents by treatment duration (*p*> 0.05). Bottom. Percentages of respondents of each endodontic program who prescribe antibiotics in the different clinical situations: 1) Irreversible pulpitis with moderate/severe symptoms. 2) Irreversible pulpitis with symptomatic apical periodontitis and moderate/severe symptoms. 3) Necrotic pulp with asymptomatic apical periodontitis, no swelling, and no or mild symptoms. 4) Necrotic pulp with symptomatic apical periodontitis, no swelling, and moderate/severe symptoms. 5) Necrotic pulp with asymptomatic apical periodontitis, sinus tracts and no/mild symptoms. 6) Necrotic pulp with symptomatic apical periodontitis, swelling, and moderate to severe symptoms. * *p*< 0.05.

The average duration proposed for antibiotic therapy was 6.8 ± 1.2 days, 7 days being the more frequently selected duration (81%) (Fig. 2, top). Only 7.5% of respondents prescribed antibiotics for more than 7 days. There were no significant differences amongst the dentists of the five Dental Schools included in the study (*p* > 0.05).

-Preferred antibiotics

Amoxicillin was chosen by all respondents as first choice antibiotic in patients with no medical allergies ( Table 2), either alone (40.3%) or associated to clavulanic acid (59.7%). Amoxicillin 875 mg / clavulanic 125 mg was the preferred first choice antibiotic for almost half of the respondents (49.3%), whereas 18%, 15%, 7.5% and 6% of the dentists selected amoxicillin 500 mg, amoxicillin 750 mg, amoxicillin 1 g, and amoxicillin/clavulanic acid 500/125 mg, respectively. There were significant differences amongst the five dental schools (*p* = 0.004). Dentists attending the endodontic program in Santiago prescribed amoxicillin associated to clavulanic acid with a frequency significantly higher than those of Barcelona (*p* < 0.05), Valencia (*p* < 0.01) and Zaragoza (*p* < 0.05). No dentists selected clindamycin, azithromycin or metronidazole-spiramycin as first choice antibiotic for non-allergic patients.

**Table 2:**
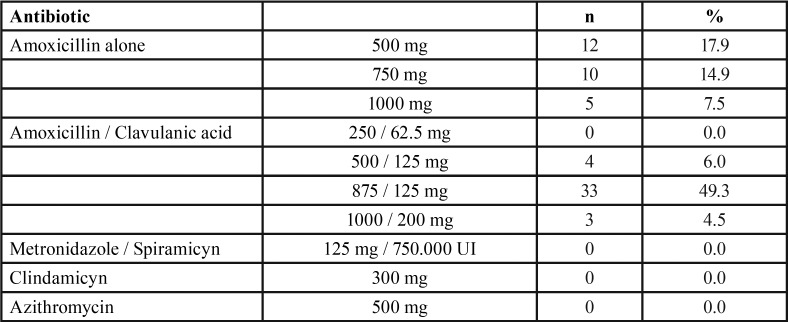
Antibiotic preference in patients with no medical allergies.

For penicillin allergic patients, the greatest majority of the respondents (72%) selected clindamycin 300 mg, and 28% selected azithromycin ( Table 3). There were also significant differences amongst the five dental schools (*p* = 0.0001). Dentists attending the endodontic program in Barcelona prescribed azithromycin with a frequency (77%) significantly higher than those of Santiago (0%, *p* < 0.01), Sevilla (16%, *p* < 0.01), Valencia (17%, *p* < 0.01) and Zaragoza (36.4%, *p* < 0.05).

**Table 3:**
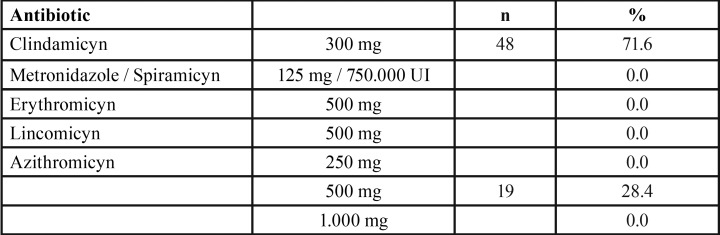
Antibiotic preference in patients with medical allergies.

-Antibiotic prescription in each clinical situation

The percentage of dentists who would prescribe antibiotics for each pulpal and periapical situation are listed in  Table 4. Twelve percent of respondents would prescribe antibiotics in patients diagnosed with irreversible pulpitis with moderate/severe symptoms. There were significant differences amongst the dental schools (*p* = 0.012). Dentists attending the endodontic program in Barcelona prescribed antibiotics with a frequency (38.5%) significantly higher than those of Santiago (0%, *p* < 0.05) and Zaragoza (0%, *p* < 0.05) (Fig. 2, bottom).

**Table 4:**
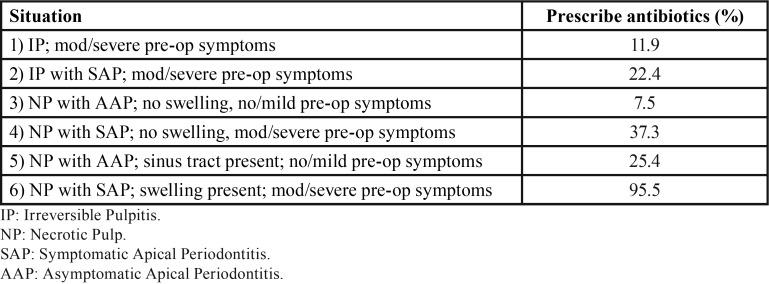
Clinical situations in which antibiotics are prescribed.

In the second clinical situation, irreversible pulpitis with symptomatic apical periodontitis and moderate/severe symptoms, 22% of respondents would prescribe antibiotics. The percentages of dentists who would prescribe AB in this clinical situation were very high in Valencia (42.9%), Barcelona (38.5%) and Santiago (33.3%) (Fig. 2, bottom) (*p* = 0.018), but there were no significant differences between the faculties compared two to two.

In the third clinical situation, asymptomatic apical periodontitis, no swelling, and no or mild symptoms, only 7.5% of respondents would have prescribed antibiotics. However, a high percentage of dentists from Barcelona (23.1%) would prescribe AB in this clinical situation, without there being differences between the five dental schools (*p* = 0.18) (Fig. 2, bottom).

In the situation of necrotic pulp, symptomatic apical periodontitis, moderate/severe symptoms but no swelling, 37% of dentists prescribed antibiotics. There were significant differences amongst the five dental schools (*p* = 0.015), being the percentages of respondents who would prescribe AB in this clinical situation significantly higher in the dentists of Barcelona (61.5%) and Valencia (57.1%) compared to those of Zaragoza (6.7%) (*p* < 0.05) (Fig. 2, bottom).

For the fifth scenario, necrotic pulp, asymptomatic apical periodontitis, but with the presence of a sinus tract, 25% of dentists would prescribed antibiotics. Again, there were significant differences amongst the universities (*p* = 0.0007) (Fig. 2, bottom). Dentists attending the endodontic program in Barcelona (46.2%) and Valencia (57.1%) prescribed antibiotics with a frequency significantly higher than those of Santiago (0%, *p* < 0.01) and Sevilla (5%, *p* < 0.05).

Finally, in the clinical situation of a necrotic pulp, symptomatic apical periodontitis, swelling, and other moderate/severe symptoms, 96% of dentists prescribed antibiotics. There were significant differences amongst the dental schools (*p* = 0.005) (Fig. 2, bottom). Dentists attending the endodontic program in Santiago prescribed antibiotics with a frequency (66.7%) significantly lower compared to those of Zaragoza (100%, *p* < 0.01), Sevilla (100%, *p* < 0.01), Valencia (100%, *p* < 0.01) and Barcelona (92.3%, *p* < 0.05).

## Discussion

The objective of this study was to analyse the antibiotic prescription habits of Spanish dentists attending postgraduate training in Endodontics when treating endodontic infections. To the best of our knowledge, this is the first survey analysing the pattern of antibiotic prescription of dentists attending specialization programs in Endodontics. The questions that were included in the survey, as well as the different endodontic clinical situations proposed, were based on the previously published surveys in the United States (21,22) and Spain (9,10,15).

-Main result

The survey responses demonstrate that all the respondents selected the proper antibiotic (amoxicillin) for the treatment of endodontic infections. However, still some of them indicated antibiotics inappropriately for the treatment of pulpitis and apical periodontitis.

-Choice of subjects

The population sampled was Spanish dentists attending specialization programs in Endodontics. Dentists were recruited from five endodontic postgraduate programs developed in five different Spanish Dental Schools, from different regions of the country. The percentage of students included in the study and the overall response rate (91%) was high. The sample (n = 67) can be considered representative of the Spanish dentists attending endodontic programs. Other published surveys, conducted under equivalent conditions, have also shown very high response rates (23,24,15).

The sample size (n = 67) is smaller than those of previous publications (15,23-25), which is explained by the smaller size of the population from which it has been extracted, i.e. the dentists attending endodontic formation. In previously published surveys in Spain (15) women were the majority of respondents (71%), reflecting the feminization of the profession of dentist in Spain. On the contrary, in this survey 51% and 49% of respondents were males and females, respectively. This could indicate that Endodontics is a field of greater gender equality in dentistry.

-First choice antibiotics and treatment duration 

Dentist proposed for antibiotic therapy a duration of 6.8 ± 1.2 days, being 7 days the mode (81%), without significant differences amongst the universities enrolled in the study. Similar results have been reported in the surveys carried out amongst Spanish endodontists (9) and oral surgeons (10), as well as in the recently published survey amongst dental students (15), and other surveys carried out in other countries (14). If the cause is treated or eliminated, most of endodontic infections resolve in three to seven days (19,26).

All respondents selected amoxicillin as the first choice antibiotic for non-allergic patients. This is in agreement with the previous results of surveys carried out amongst the members of the Spanish Endodontics Society, who selected amoxicillin as the first-choice antibiotic (82%) (9), and with the members of the Spanish Oral Surgery Society, who also chose amoxicillin (95%) (10).

Surveys carried out in other European countries (11,12,14,27,28) also found amoxicillin as the first choice antibiotic selected by dentists for the treatment of apical periodontitis. Amoxicillin is a good antibiotic for pulp-periapical disease because its great antimicrobial activity against the microbiota responsible of these pathologies. However, taking into account the development of β-lactamase producing bacteria, the combination of amoxicillin with a β-lactamase inhibitor, such as clavulanic acid (co-amoxiclav), is recommended (29-32).

For β-lactam allergic patients, the first choice antibiotic was clindamycin 300 mg (72%), followed by azithromicyn (28%). These results are in accordance with the results of the surveys conducted previously in Spain amongst endodontists (63% clindamycin) (9) and oral surgeons (65% clindamycin) (10).

-Prescription of antibiotics in the suggested clinical situations and comparison with previous surveys

The results of the present study demonstrate that postgraduate training in Endodontics improves dentists’ knowledge of indications of antibiotics in the treatment of endodontic infections. The pattern of antibiotic prescription of dentists attending specialization programs on Endodontics, found in this study, fits better to the correct guidelines (7) than those of dentists without specialized formation on Endodontics (9,10) and those of dental students (15). This is confirmed when analysing the answers given to each of the clinical situations that were proposed to them.

The two first clinical situations are both cases of irreversible pulpitis, the pulp is still vital, with no signs of systemic involvement. Therefore, antibiotics are not indicated in these situations (6-8,33). However, 12% and 22% of dentists, respectively, prescribed antibiotics. But these percentages are low compared to 29% and 63% of final year students who would prescribe antibiotics in the same cases (15), as well as compared to the pattern of prescription of Spanish dentists without postgraduate training in Endodontics (10).

The third clinical situation is a necrotic pulp in a healthy patient with no signs of systemic involvement. Neither in this case are antibiotics indicated (7,14), what was well known by the surveyed dentists, who mostly (92%) did not prescribe antibiotics. On the contrary, the surveys carried out in 2009 (9) and 2010 (10) amongst Spanish dentists showed that 14%-31% prescribed antibiotics in this situation. Taking into account that, recently, 60% of final years Spanish dental students responded that they would indicate antibiotics in this situation (15), the results of the present survey suggest that specialized training in Endodontics improves the knowledge of Spanish dentists about the indications of antibiotics in the treatment of endodontic infections.

Neither the fourth nor the fifth case, necrotic pulp with symptomatic apical periodontitis with moderate/severe symptoms and no swelling, and necrotic pulp with asymptomatic apical periodontitis with sinus tract, respectively, requires antibiotics. Nonsurgical root canal treatment and analgesics are sufficient (6,8). However, 37% and 25% of respondent dentists prescribe antibiotics in the fourth and fifth clinical situation, respectively. Previous studies have found higher percentages of antibiotics prescription in the fourth (53%-71%) and the fifth (21%-60%) situations (9,10). Moreover, surveys carried out amongst dentists in other European countries and in other continents (14), have reported higher percentages of antibiotic prescription in this two situations. Again, the present results demonstrate that Spanish dentists with specialized training in Endodontics have a better knowledge about the indications of antibiotics in Endodontics.

Finally, the last clinical scenario, a necrotic pulp with symptomatic apical periodontitis and systemic involvement (swelling and moderate/severe symptoms), indicates antibiotics in addition to root canal treatment, incision and drainage (6-8). Effectively, 96% of respondent dentists treated this clinical situation with antibiotics. In this case, systemic antibiotics are correctly indicated as an adjunct to endodontic clinical treatment in order to prevent the spread of infection (7,8,34).

The comparison of the results of this survey (dentists of postgraduate training programs in endodontics) with the results of previous surveys carried out on undergraduate students (15) or general dentists without specialized training in endodontics (10-14), show that their knowledge about the indications of antibiotics in Endodontics has been improved (9-16). However, the percentages of dentists who would prescribe antibiotics in situations where they are not indicated are still high.

-Differences amongst training programs

Particularly striking is the differences observed between endodontic training programs: dentists trained in Barcelona and Valencia have high percentages of antibiotic over-prescription. On the contrary, dentists from the endodontic programs in Sevilla and Zaragoza show lower percentages of antibiotic prescription.

The differences observed between the programs could be explained by the different importance given, within each of the programs, to the medical aspects of endodontics. Some endodontic programs focus, perhaps too much, on the technical and technological aspects of endodontics, dedicating less importance to its basic, medical and pharmacological aspects. Possibly, the formation and the research field of the professors of each program have a great influence on this.

Another factor that may explain the high percentage of prescription of antibiotics observed in some of the programs may be the type of population served. The pressure imposed by the community and the unreasonable demand for antibiotics by patients is a key factor in over-prescription, and may vary from one region to another.

The European Society of Endodontology published the guidelines for Accreditation of postgraduate speciality training programs in Endodontology (1) with the aim of achieving uniformity in Endodontics teaching programs in Europe. Certainly, it should be achieved that the endodontic training programs taught in the different European countries have similar and complete contents. However, controlling that this is so is very difficult.

Although the minimum criteria for training specialists in Endodontology within Europe remark that endodontic trainees must have an understanding of the relevant aspects of the clinical pharmacology and therapeutics in relation to the treatment of endodontic infections (1), the results of this study show that it may be neglecting in some programs. This type of surveys can be a good instrument to detect aspects of endodontic training that should be improved. In fact, the results of this study have already helped some of the endodontic programs that have participated in it to introduce changes in their programming that, with certainty, will improve the preparation of the dentists involved in them.

## Conclusions

The results of this study show that postgraduate training in Endodontics provides greater awareness of the correct indications of antibiotics. Dentists who have received specialized training in Endodontics have a prescription pattern of antibiotics more adjusted to the guidelines recommended by international organizations and by scientific societies. However, there is still a significant percentage of dentists with specialized training in Endodontics who prescribe antibiotics in clinical situations where they are not indicated. Therefore, campaigns must still be carried out to promote the appropriate use of systemic antibiotics in Endodontics, preventing their misuse and protecting patients against unnecessary antibiotics.
